# Validation of the i-STAT system for the analysis of blood parameters in fish

**DOI:** 10.1093/conphys/cou037

**Published:** 2014-09-03

**Authors:** T. S. Harter, R. B. Shartau, C. J. Brauner, A. P. Farrell

**Affiliations:** Department of Zoology, University of British Columbia, 6270 University Boulevard, Vancouver, BC, Canada V6T 1Z4

**Keywords:** Blood oxygen tension, blood pH, haematocrit, portable clinical analyser, rainbow trout

## Abstract

Portable clinical analysers, such as the i-STAT system, are increasingly being used for blood analysis in animal ecology and physiology because of their portability and easy operation. Although originally conceived for clinical application and to replace robust but lengthy techniques, researchers have extended the use of the i-STAT system outside of humans and even to poikilothermic fish, with only limited validation. The present study analysed a range of blood parameters [pH, haematocrit (Hct), haemoglobin (Hb), HCO_3_^−^, partial pressure of CO_2_ (*P*CO_2_), partial pressure of O_2_ (*P*O_2_), Hb saturation (sO_2_) and Na^+^ concentration] in a model teleost fish (rainbow trout, *Oncorhynchus mykiss*) using the i-STAT system (CG8+ cartridges) and established laboratory techniques. This methodological comparison was performed at two temperatures (10 and 20°C), two haematocrits (low and high) and three *P*CO_2_ levels (0.5, 1.0 and 1.5%). Our results indicate that pH was measured accurately with the i-STAT system over a physiological pH range and using the i-STAT temperature correction. Haematocrit was consistently underestimated by the i-STAT, while the measurements of Na^+^, *P*CO_2_, HCO_3_^−^ and *P*O_2_ were variably inaccurate over the range of values typically found in fish. The algorithm that the i-STAT uses to calculate sO_2_ did not yield meaningful results on rainbow trout blood. Application of conversion factors to correct i-STAT measurements is not recommended, due to significant effects of temperature, Hct and *P*CO_2_ on the measurement errors and complex interactions may exist. In conclusion, the i-STAT system can easily generate fast results from rainbow trout whole blood, but many are inaccurate values.

## Abstract (precis)

The i-STAT system, a portable clinical analyzer, is increasingly being used to assess blood parameters in fish. This study validated the i-STAT system on rainbow trout blood under a broad range of conditions. Results indicate that the i-STAT is not an appropriate tool for assessing most blood parameters in rainbow trout.

## Introduction

The analysis of blood parameters plays a key role in determining an organism's health- and/or physiological status. Thus, blood sampling and analysis are routinely performed in human and veterinary medicine, as well as more broadly in physiological research, including the growing fields of wildlife surveillance and conservation physiology (Cooke *et al.*, 2013). While adequate blood sampling is now easily accomplished using cannulation and arterial puncture or venipuncture techniques, analysis typically requires sophisticated laboratory equipment, trained personnel and a considerable time investment. Some parameters, such as plasma ion and haemoglobin (Hb) concentrations, can be assessed after freezing (a prerequisite for many field studies in biology), but other blood parameters (e.g. acid–base status and blood gases) need to be measured immediately after collection for maximal accuracy. This requirement sets boundaries for the blood parameters that can be assessed using conventional techniques in remote locations and has been a considerable constraint for remote patient care and for field research on the physiology of non-domesticated animal taxa. However, the recent advances in medical analytical instruments have allowed the development of portable devices capable of accurately assessing a variety of relevant blood parameters; one of the most widely used systems today is the i-STAT^®^ (Abbot Point of Care Inc., Princeton, NJ, USA).

The i-STAT is a rugged, light-weight, battery-powered, handheld device that allows simultaneous measurement of many blood parameters in minutes and using merely 100 μl of whole blood. Owing to these undisputed advantages over conventional analytical techniques, the i-STAT system is gaining popularity in biological research, for the analysis of blood on site and on a wide range of species, including mammals (e.g. Stockard *et al.*, 2007), birds (e.g. Paula *et al.*, 2008), reptiles (e.g. Harms *et al.*, 2003) and a large number of fish species (see Stoot *et al.*, 2014). In fact, the measurement of blood gases and acid–base status of many wild animals became possible only after the introduction of portable clinical analysers, such as the i-STAT system; and still today, it is often the only viable method available to researchers working in the field who are interested in measuring these important physiological parameters.

However, it is of note that the i-STAT system was originally conceived for medical application and that the measurement and especially the calculation of many parameters are based on constants and algorithms derived for human blood. It seems unlikely that a system designed for a single species could account for differences in blood characteristics between species and even taxonomic classes. Perhaps of greatest concern should be the effect of temperature (if different from 37°C, as in all ectothermic animals) on the accurate assessment of blood acid–base status and blood gases, because complex interactions between these factors exist upon closed system temperature changes (Malte *et al.*, 2014). In addition, the measurement of haematocrit (Hct) may be subject to bias, because some of the studied species, such as birds, reptiles and fish, have nucleated red blood cells (RBCs) that will differ substantially from human RBCs in cell size and shape, metabolic activity, Hb content and Hb isoforms (Nikinmaa, 1990). The analysis of blood parameters in fishes is particularly challenging, because some species have unique Hb characteristics, such as a strong Bohr/Haldane effect (Bohr *et al.*, 1904; Brauner and Jensen, 1999) or even a Root effect (Root, 1931; Brittain, 2005), which respond to changes in blood gases and acid–base status; these factors cannot be accounted for by algorithms designed for human blood.

Despite these limitations, Stoot *et al.* (2014) identified 27 studies in the last decade that have used the i-STAT system to measure blood parameters in several elasmobranch and teleost species and in a variety of environmental and physiological conditions (Kojima *et al.*, 2004; Harrenstien *et al.*, 2005; Foss *et al.*, 2006, 2007, 2012; Choi *et al.*, 2007; Eliason *et al.*, 2007; Mandelman and Farrington, 2007a, b; Olsvik *et al.*, 2007; Suski *et al.*, 2007; Brill *et al.*, 2008; Imsland *et al.*, 2008; Petri *et al.*, 2008; Remen *et al.*, 2008; Mandelman and Skomal, 2009; DiMaggio *et al.*, 2010; Gallagher *et al.*, 2010; Meland *et al.*, 2010; Olsvik *et al.*, 2010; Brooks *et al.*, 2011, 2012; Cicia *et al.*, 2012; Frick *et al.*, 2012; Hyatt *et al.*, 2012; Naples *et al.*, 2012; Roth and Rotabakk, 2012). While the authors of some of these studies recognized that certain blood parameters were not accurate, they argued that relative differences were still meaningful and worthwhile assessing (e.g. Mandelman and Farrington, 2007b; Cooke *et al.*, 2008; Gallagher *et al.*, 2010). Moreover, to the best of our knowledge, only three studies on fish have validated i-STAT results with conventional laboratory techniques (Harrenstien *et al.*, 2005; DiMaggio *et al.*, 2010; Gallagher *et al.*, 2010). DiMaggio *et al.* (2010; validation for Hct, sodium, potassium and chloride) concluded that the i-STAT is not a reliable tool to assess Hct or plasma ions in seminole killifish (*Fundulus seminolis*). Both Harrenstien *et al.* [2005; validation for urea nitrogen, glucose, sodium, potassium, chloride, pH, partial pressure of CO_2_ (*P*CO_2_), total CO_2_ (TCO_2_), HCO_3_^−^, base excess, Hct and Hb] and Gallagher *et al.* [2010; validation for pH, *P*CO_2_, partial pressure of O_2_ (*P*O_2_), Hb saturation (sO_2_) and lactate] reported significant differences between measurements performed with the i-STAT and those using conventional techniques on two elasmobranch species and two rockfish species, respectively. However, latter authors concluded that in the tested conditions and applying appropriate conversion factors, the i-STAT system was a useful tool for measuring blood parameters. Whether these conversion factors will hold over a broader range of conditions has not been addressed previously; therefore, extrapolation of the determined conversion factors beyond the tested conditions is considered problematic.

As outlined above, the following three factors are most likely to bias i-STAT measurements: (i) temperature; (ii) differences in nucleated vs. non-nucleated RBC, hence Hct; and (iii) blood gas concentrations and acid–base status (that vary with *P*CO_2_), especially in teleosts that have a strong Bohr/Root shift (such as rainbow trout, *Oncorhynchus mykiss*). In our view, addressing these potentially confounding factors is important for a thorough validation of the i-STAT system for its use in the analysis of fish blood.

The aim of the present study was to validate the i-STAT system for the measurement of blood parameters in the model teleost, rainbow trout, over a broader range of conditions. Our choice of parameters was based on previous studies, most of which used the i-STAT system to measure pH, *P*CO_2_ and *P*O_2_. In addition, we deemed necessary a validation of the important blood parameters Hct and Hb, because of the fundamental differences between fish and mammalian RBCs and between fish Hb and that of other vertebrates. Therefore, blood samples were experimentally adjusted to one of two Hct levels (low, 20%; and high, 30%) and equilibrated in tonometers at two temperatures (10 and 20°C) and to three levels of *P*CO_2_ (0.5, 1.0 and 1.5%). We measured whole-blood Hct, Hb concentration, sO_2_ and pH and plasma *P*CO_2_, HCO_3_^−^ and Na^+^ using the i-STAT system and compared these values with those measured using conventional techniques. While the i-STAT system offers tremendous benefits for field studies in experimental biology, this potential can be realized only after a thorough technical validation of the obtained measurements. Therefore, the present results provide guidelines for an appropriate implementation of the i-STAT system into future research on fish.

## Materials and methods

### Animals and housing

Rainbow trout, *Oncorhynchus mykiss* Walbaum 1792 (300–600 g), were obtained from Spring Valley Trout Farm (Langley, British Columbia, Canada) and were held at the University of British Columbia aquatic facilities in 4000 l tanks supplied with flow-through dechlorinated municipal tap water (Vancouver, BC, Canada), under a natural photoperiod, at 12°C. Animals were fed three times a week to satiation using commercial trout pellets (Skretting, Orient 4-0, Vancouver, British Columbia, Canada), but feeding was suspended 24 h before blood collection. All procedures were strictly according to the guidelines specified by the Canadian Council on Animal Care (UBC protocol no. A07-0080).

### Blood collection

In preparation for surgery, fish were anaesthetized for 2 min in 0.5 g l^−1^ MS222 (tricaine methanosulfonate) buffered with sodium bicarbonate (1 g l^−1^ NaHCO_3_^−^). When unresponsive, animals were placed on a surgery table, and their gills were continuously perfused with aerated water (12°C) containing a maintenance anaesthetic dose (0.2 g l^−1^ buffered MS222). An indwelling cannula (PE50) was chronically implanted into the dorsal aorta according to the method described by Soivio *et al.* (1975). After surgery, the gills were perfused with cooled, aerated water until responsiveness was regained, and animals were transferred to individual flow-through Perspex boxes supplied with aerated water at 12°C. The cannula was flushed with heparinized Cortland saline (10 i.u. ml^−1^, lithium heparin; Sigma H0878), and animals were allowed to recover from surgery for 24 h. Blood samples of up to 5 ml were taken from undisturbed fish and were drawn from a cannula into a pre-heparinized syringe. Blood was pooled from up to six fish and kept on ice until analysis.

Haematocrit was measured in triplicate and then standardized to a predetermined Hct by either removing or adding plasma that had previously been obtained by caudal puncture (immediately frozen at −80°C). A 3 ml aliquot of blood was loaded into each of six Eschweiler tonometers placed in a thermostated water bath and equilibrated with a water-saturated gas mixture. All experimental conditions were chosen to mimic the *in vivo* status of rainbow trout venous blood, as described by Rummer (2010). Blood samples in tonometers were equilibrated to treatment conditions for 1 h before analysis using custom-mixed gases (O_2_, CO_2_ and N_2_) from a DIGAMIX 275 6KM 422 Woesthoff pump (Bochum, Germany).

### Experimental design

The i-STAT system was validated over a broad range of conditions for three factors (Hct, temperature and *P*CO_2_). Six replicate samples (*n* = 6) were run for each combination of factors, and the experimental unit was a single tonometer containing blood pooled from up to six fish. This design resulted in a total of 72 blood samples, which represented blood pooled from 36 donor fish.

Haematocrit was set to either low (∼20%) or high (∼30%); these values are within the normal range for rainbow trout (Gallaugher and Farrell, 1998). Two temperatures were tested, either 10 or 20°C, and these temperatures were used during conventional blood analyses. The *P*CO_2_ was tested at three levels by setting the composition of the equilibration gas mixture during tonometry to 0.5 (∼3.8 mmHg), 1.0 (∼7.5 mmHg) or 1.5% (∼11.3 mmHg), which mimicked the *in vivo* venous *P*CO_2_ tension range of rainbow trout (Rummer, 2010). The *P*O_2_ was nominally set to 6% (∼45.9 mmHg) to approximate Hb O_2_ saturations of 50–100%. Nitrogen was used as a balance gas.

### Sampling protocol

The six tonometers were sampled sequentially after equilibration intervals of 45 min using heparinized, gas-tight Hamilton syringes of appropriate volume. Each time, three subsamples were taken from a tonometer. A 90 μl subsample was immediately loaded into the i-STAT cartridge; a 20 μl subsample was used for the analysis of total O_2_ (TO_2_); and a subsample of 500 μl was removed for the analysis of pH, Hct, Hb concentration, TCO_2_ and Na^+^ concentration. First, whole-blood pH was measured at treatment temperature, Hct was measured in triplicate and samples for the measurement of Hb concentration were stored at 4°C (analysis was within 48 h). The remainder of the blood sample was centrifuged (10 000 *g* for 2 min); 20 μl of plasma were stored at −80°C for the analysis of Na^+^ concentration, and TCO_2_ was measured on the remaining plasma.

### Analysis

Measurements were performed using the VetScan i-STAT 1 System (SN:704534-C; software version JAMS 135c/CLEW A26; Abaxis, Union City, CA, USA) with the i-STAT CG8+ cartridge test (ABBT-03P77-25). Cartridges were stored in the dark in their original packaging at 4°C. Before use, cartridges were allowed to equilibrate to room temperature overnight. Analysis was at room temperature, using the temperature correction function of the i-STAT system for the two experimental temperatures.

Using an established laboratory technique, Hct was measured by filling microcapillary tubes (10 μl) and centrifuging at 17 000 ***g*** for 3 min. Likewise, Hb concentration was measured in duplicate with a Beckman 640 spectrophotometer (Brea, CA, USA) using the cyanomethaemoglobin method and an extinction coefficient of 11 mmol^−1^ cm^−1^ at 540 nm. Whole-blood pH measurements were performed using a Radiometer BMS 3 Mk2 and a Radiometer acid–base analyser PHM73 (Copenhagen, Denmark) with a thermostated pH electrode at the respective treatment temperature. Whole-blood TO_2_ content was measured according to Tucker (1967). Haemoglobin saturation was calculated from TO_2_ after subtracting physically dissolved O_2_ according to Boutilier *et al.* (1984) and dividing by the theoretical maximal carrying capacity of the rinsed RBCs based upon the tetrameric Hb concentration obtained spectrophotometrically according to Tucker (1967). Total CO_2_ in the blood plasma was measured using a Corning 965 CO_2_ analyser (Corning, USA), after centrifuging whole blood for 5 min at 5000 ***g***. Plasma HCO_3_^−^ concentration and *P*CO_2_ were calculated from the plasma pH and TCO_2_ using the Henderson–Hasselbalch equation and the physicochemical parameters reported by Boutilier *et al.* (1984). Plasma Na^+^ concentration was measured after a 2000:1 dilution with milliQ de-ionized water (resistivity >18.2 μΩ cm^−1^) and using a Varian AA240FS atomic absorption spectrophotometer (Palo Alto, CA, USA).

### Data analysis

All data were analysed with RStudio v0.98.501 (RStudio Inc., Boston, MA, USA). i-STAT measurements were compared with control measurements by regression analysis using the original data. The measurement error for the i-STAT values relative to control measurements was calculated as follows: δ = (i-STAT-control)/control × 100. The δ data were then compared with control measurements either by regression analysis or by fitting a non-linear model to the data [linear, logarithmic and exponential models were compared using the Akaike information criterion (AIC), and the model with the best fit was used as representative for the data]. Normality of distribution was tested with the Shapiro–Wilk test (*P* < 0.05), and homogeneity of variances was tested with Levene's test (*P* < 0.05). The effects of temperature and Hct on δ were tested with the Welch independent samples *t*-test (*P* < 0.05, *n* = 36) and *P*CO_2_ with one-way ANOVA (*P* < 0.05, *n* = 24), using the absolute values of δ (i.e. by treating all values as positive). In some cases, this transformation led to a significant deviation of the distribution from normality, which could not be remediated by data transformation; in this situation, the effects of temperature and Hct on δ were tested with the Wilcoxon rank sum test (*P* < 0.05, *n* = 36) and *P*CO_2_ with the Kruskal–Wallis rank sum test (*P* < 0.05, *n* = 24). All data are presented as means ± SEM.

## Results

In general, we perceived the i-STAT system as very user friendly. All 72 i-STAT CG8+ cartridges used in the present study performed adequately. There was no clotting inside the cartridges or erroneous readings for other reasons. Measurements were performed on a mere 90 μl of whole blood, and all procedures could easily be carried out by a single person within a few minutes. The control measurements on the same blood parameters, in contrast, required two people working simultaneously for about 45 min per sample (excluding the measurements of Hb and Na^+^ concentrations that were performed on preserved samples at a later point in time).

### pH

**Figure 1:**
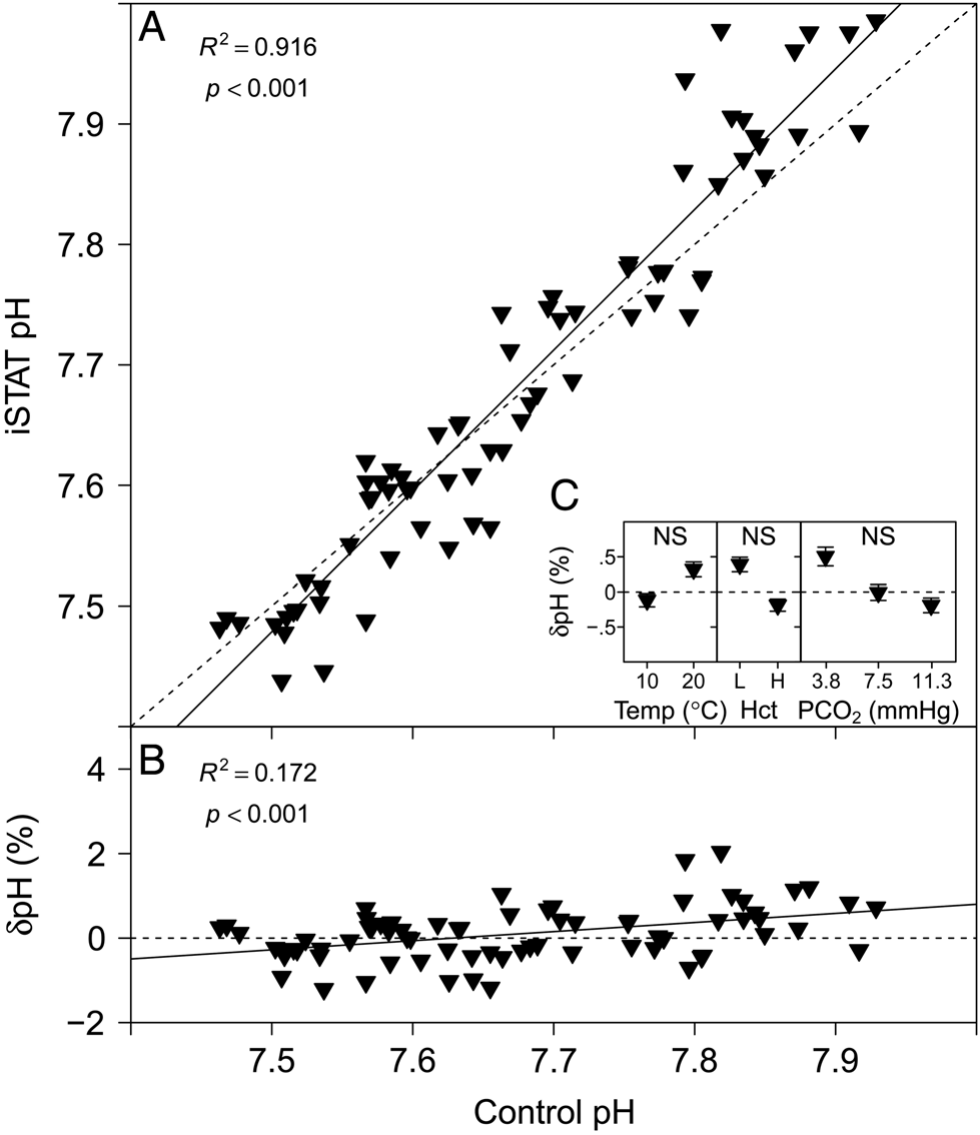
(**A**) Measurements of pH with the i-STAT system vs. control measurements of pH with a thermostated pH electrode. (**B**) The relative error of i-STAT pH measurements, δpH (expressed as a %), [calculated as: (i-STAT pH − control pH)/control pH × 100] vs. control pH. Continuous lines represent the fitted linear models and dashed lines represent the lines of identity. (**C**) Effects of temperature (in °C ), haematocrit (Hct; L, low; H, high) and partial pressure of CO_2_ (*P*CO_2_; in mmHg) on δpH (expressed as a %). Significant effects within treatments are indicated as NS for non-significant. Data are means ± SEM, and statistical analysis was performed on the absolute δpH values.

The pH measurements performed with the i-STAT system are compared with measurements made with a thermostated pH electrode in Fig. 1A. A highly significant linear relationship was found between i-STAT and control pH (Table 1). The relative error of pH measurements performed with the i-STAT, δpH (expressed as a percentage), is compared with the control pH in Fig. 1B, and the parameter estimates for the linear relationship are given in Table 1. No significant effects on δpH were detected for temperature (*P* = 0.871, *n* = 36), Hct (*P* = 0.065, *n* = 36) or *P*CO_2_ (*P* = 0.347, *n* = 24; Fig. 1C).

**Table 1:** Parameter estimates (means ± SEM), *r*^2^ and *P*-values for the relationships between i-STAT vs. control measurements and i-STAT measurement errors, δ(*x*) (expressed as a percentage), vs. control measurements (*n* = 72)

Measurement	*a*	*b*	*c*	*r*^2^	*P*-value
pH	−1.280 ± 0.322	1.168 ± 0.042		0.916	<0.001
δpH	−16.455 ± 4.171	2.157 ± 0.543		0.172	<0.001
Hct	−3.945 ± 1.008	0.803 ± 0.041		0.842	<0.001
δHct	−54.006 ± 4.009	0.723 ± 0.164		0.206	<0.001
Na^+^	135.701 ± 4.821	0.027 ± 0.037		−0.007	0.471
δNa^+^	1600 ± 1012	0.969 ± 0.006*	−19.562 ± 5.189*		
*P*CO_2_	2.076 ± 0.106	0.876 ± 0.013		0.984	<0.001
δ*P*CO_2_	111.730 ± 12.543*	0.774 ± 0.036*	−0.399 ± 3.369		
HCO_3_^−^	3.150 ± 0.491	0.836 ± 0.044		0.838	<0.001
δHCO_3_^−^	41.961 ± 4.300	−2.630 ± 0.381		0.396	<0.001
sO_2_	96.990 ± 0.316	0.033 ± 0.004		0.425	<0.001
δsO_2_	562.073 ± 9.798*	0.970 ± 0.001*	−26.055 ± 1.311*		

Abbreviations: Hb, haemoglobin; Hct, haematocrit; *P*CO_2_, partial pressure of CO_2_; *P*O_2_, partial pressure of O_2_; and sO_2_, haemoglobin saturation, δ, measurement error.

Linear relationships according to: i-STAT(*x*) = *a* + *b* × control(*x*); and δ(*x*) = *a* + *b* × control(*x*).

Non-linear relationships according to: i-STAT(*x*) = *a* × *b*^control(^^*x*^^)−^^*c*^.

*Parameter estimates in non-linear models marked with ‘*’ are statistically significant (*t*-test, *P* < 0.001).

### Haematocrit

**Figure 2:**
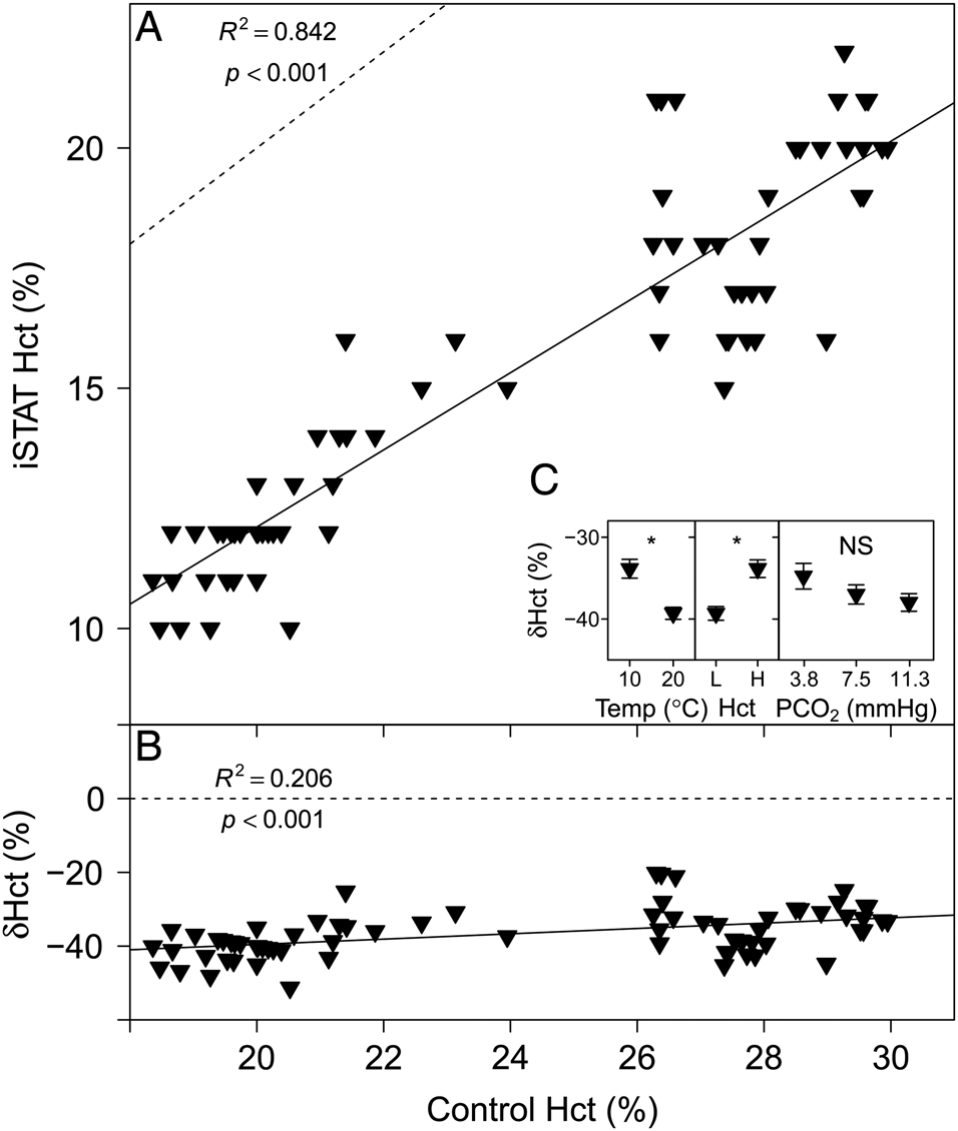
(**A**) Measurements of Hct with the i-STAT system vs. control measurements of Hct in microcapillary tubes. (**B**) The relative error of i-STAT Hct measurements, δHct (expressed as a %), [calculated as: (i-STAT Hct − control Hct)/control Hct × 100] vs. control Hct. Continuous lines represent the fitted linear models and dashed lines represent the lines of identity. (**C**) Effects of temperature (in °C ), Hct (L, low; H, high) and *P*CO_2_ (in mmHg) on δHct (expressed as a %). Significant effects within treatments are indicated as NS for non-significant and ‘*’ at the *P* < 0.05 level. Data are means ± SEM, and statistical analysis was performed on the absolute δHct values.

The Hct measurements performed with the i-STAT system are compared with measurements in microcapillary tubes in Fig. 2A. A highly significant linear relationship was found between i-STAT and control Hct measurements (Table 1). The relative error of Hct measurements performed with the i-STAT, δHct (expressed as a percentage), compared with control Hct was a constant underestimate (Fig. 2B), and the parameter estimates for the linear relationship are given in Table 1. Temperature (*P* < 0.001) and Hct (*P* < 0.001) had a significant effect on δHct, but no significant effect of *P*CO_2_ on δHct was detected (*P* = 0.204; Fig. 2C).

### Sodium

**Figure 3:**
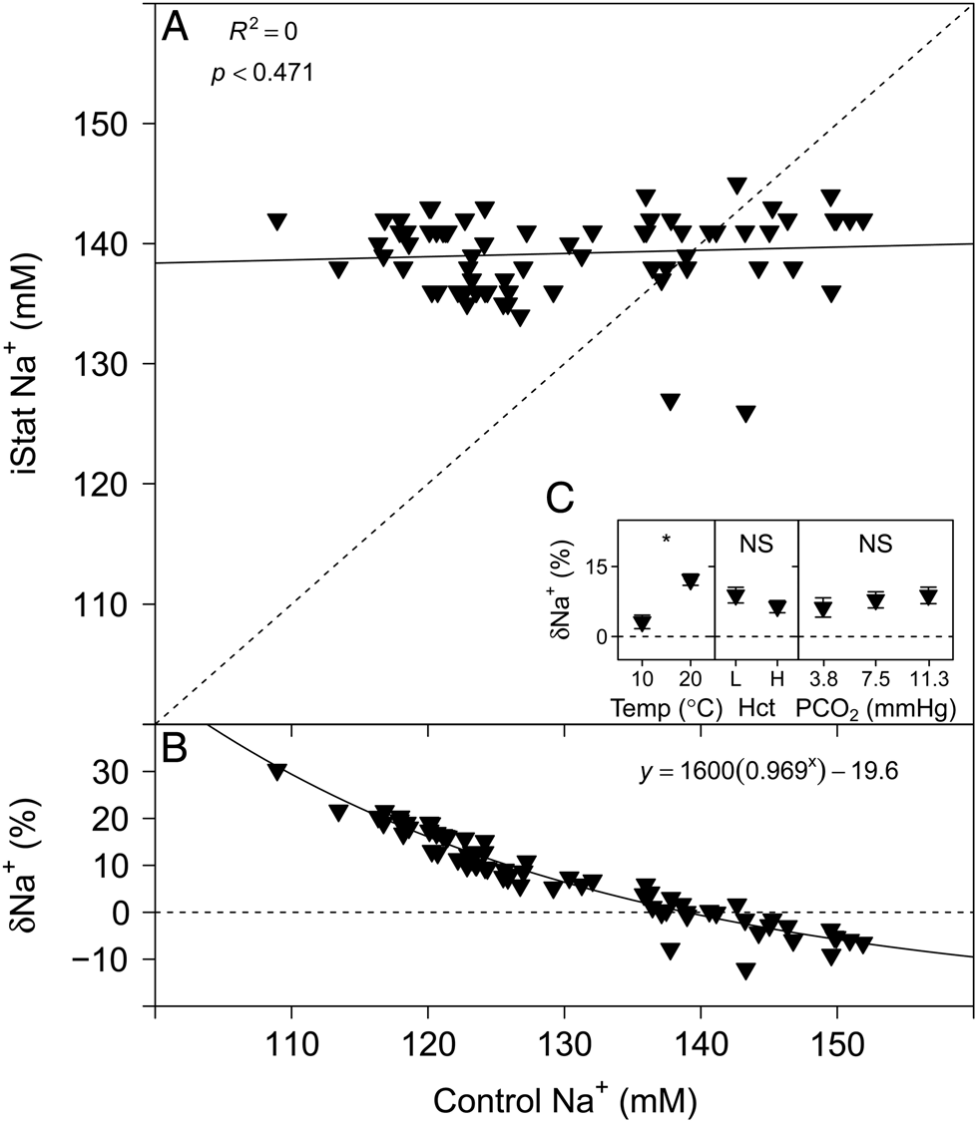
(**A**) Measurements of Na^+^ with the i-STAT system vs. control measurements of Na^+^ with a flame photometer. (**B**) The relative error of i-STAT Na^+^ measurements, δNa^+^ (expressed as a %), [calculated as: (i-STAT Hct − control Hct)/control Hct × 100] vs. control Hct. Continuous lines represent the fitted models and dashed lines represent the lines of identity. (**C**) Effects of temperature (in °C ), Hct (L, low; H, high) and *P*CO_2_ (in mmHg) on δNa^+^ (expressed as a %). Significant effects within treatments are indicated as NS for non-significant and ‘*’ at the *P* < 0.05 level. Data are means ± SEM, and statistical analysis was performed on the absolute δNa^+^ values.

The Na^+^ measurements performed with the i-STAT are compared with measurements made with a flame photometer in Fig. 3A. No evident relationship was detected between i-STAT Na^+^ and control Na^+^ (Table 1). The relative error of Na^+^ measurements performed with the i-STAT, δNa^+^ (expressed as a percentage), is compared with control Na^+^ in Fig. 3B and was best described by an exponential model (linear AIC, 358.24; logarithmic AIC, 350.46; and exponential AIC, 339.83), with the parameter estimates given in Table 1. Temperature (*P* < 0.001) had a significant effect on δNa^+^, but no significant effects of Hct (*P* = 0.054) or *P*CO_2_ on δNa^+^ were detected (*P* = 0.997; Fig. 3C).

### Partial pressure of CO_**2**_

**Figure 4:**
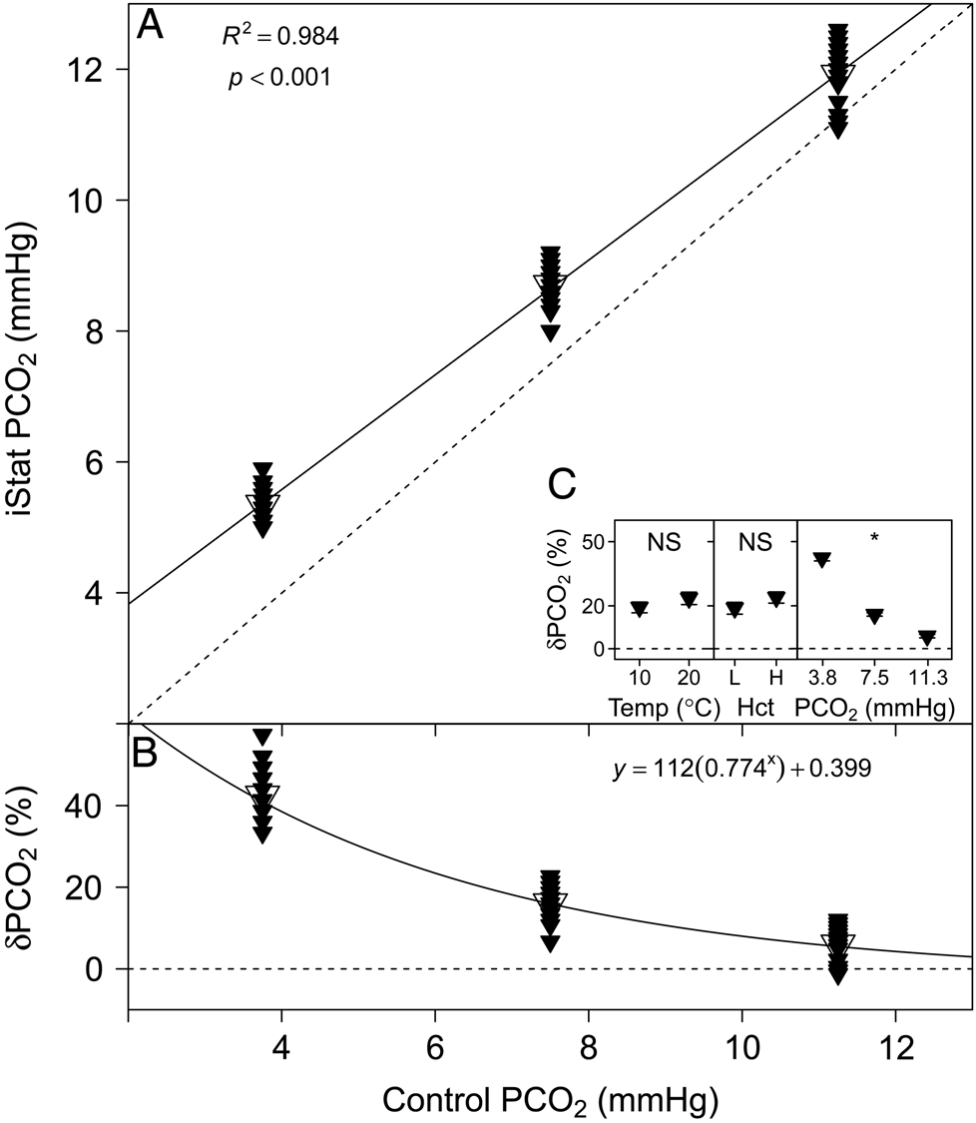
(**A**) Measurements of *P*CO_2_ with the i-STAT system vs. set *P*CO_2_. Mean values are indicated by the larger, open symbols. (**B**) The relative error of i-STAT *P*CO_2_ measurements, δ*P*CO_2_ (expressed as a % ), [calculated as: (i-STAT *P*CO_2_ − control *P*CO_2_)/control *P*CO_2_ × 100] vs. control *P*CO_2_. Continuous lines represent the fitted models and dashed lines represent the lines of identity. (**C**) Effects of temperature (in °C ), Hct (L, low; H, high) and *P*CO_2_ (in mmHg) on δ*P*CO_2_ (expressed as a %). Significant effects within treatments are indicated as NS for non-significant and ‘*’ at the *P* < 0.05 level. Data are means ± SEM, and statistical analysis was performed on the absolute δ*P*CO_2_ values.

The *P*CO_2_ measurements performed with the i-STAT are compared with the set tonometer values in Fig. 4A. A highly significant linear relationship was found between i-STAT and control *P*CO_2_ (Table 1). The relative error of *P*CO_2_ measurements performed with the i-STAT, δ*P*CO_2_ (expressed as a percentage), is compared with control *P*CO_2_ in Fig. 4B and was best described by an exponential model (linear AIC, 472.95; logarithmic AIC, 445.18; and exponential AIC, 440.02), with the parameter estimates given in Table 1. Partial pressure of CO_2_ had a significant effect (*P* < 0.001) on δ*P*CO_2_, but no significant effects of temperature (*P* = 0.260) or Hct were detected (*P* = 0.067; Fig. 4C).

### Bicarbonate

**Figure 5:**
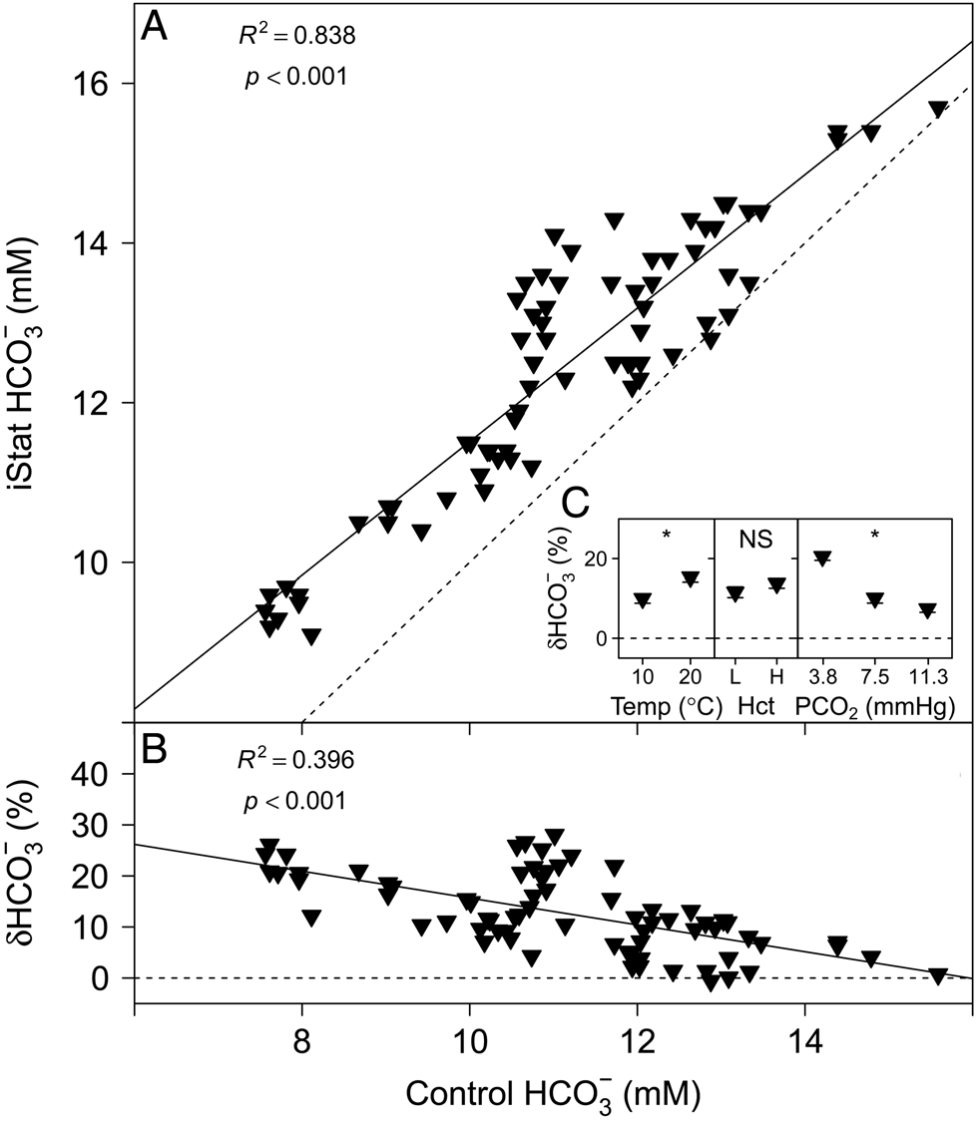
(**A**) Measurements of HCO_3_^−^ with the i-STAT system vs. control HCO_3_^−^ calculated from total CO_2_ and pH. (**B**) The relative error of i-STAT HCO_3_^−^ measurements, δHCO_3_^−^ (%), [calculated as: (i-STAT HCO_3_^−^ − control HCO_3_^−^)/control HCO_3_^−^ × 100] vs. control HCO_3_^−^. Continuous lines represent the fitted linear models and dashed lines represent the lines of identity. (**C**) Effects of temperature (in °C ), Hct (L, low; H, high) and *P*CO_2_ (in mmHg) on δHCO_3_^−^ (expressed as a %). Significant effects within treatments are indicated as NS for non-significant and ‘*’ at the *P* < 0.05 level. Data are means ± SEM, and statistical analysis was performed on the absolute δHCO_3_^−^ values.

The HCO_3_^−^ measurements with the i-STAT are compared with conventional laboratory techniques in Fig. 5A. A highly significant linear relationship was found between i-STAT and control HCO_3_^−^ measurements (Table 1). The relative error of HCO_3_^−^ measurements performed with the i-STAT, δHCO_3_^−^ (expressed as a percentage), is compared with control HCO_3_^−^ in Fig. 5B, and the parameter estimates for the linear relationship are given in Table 1. Temperature (*P* = 0.002) and *P*CO_2_ (*P* < 0.001) had significant effects on δHCO_3_^−^, but no significant effect of Hct on δHCO_3_^−^ was detected (*P* = 0.232; Fig. 5C).

### Partial pressure of O_**2**_

All treatments were measured at a *P*O_2_ of 45.9 mmHg (∼6% O_2_); therefore, the effect of *P*O_2_ on i-STAT *P*O_2_ measurements was not assessed and the results are not presented in a figure. The overall mean of i-STAT *P*O_2_ measurements was 77.9 ± 4.0 mmHg and the mean relative error of measurements, δ*P*O_2_ (expressed as a percentage), was 69.6 ± 8.6. A significant effect of temperature (*P* < 0.001) on δ*P*O_2_ was detected, and mean values were 121.3 ± 11.3 and 17.9 ± 4.4 at 10 and 20°C, respectively. Also *P*CO_2_ had a significant effect (*P* < 0.001) on δ*P*O_2_, and mean values were 121.6 ± 15.9, 67.1 ± 12.4 and 20.1 ± 7.3% at 3.8, 7.5 and 11.3 mmHg *P*CO_2_, respectively. Haematocrit did not have a significant effect on δ*P*O_2_ (*P* = 0.258).

### Haemoglobin saturation

**Figure 6:**
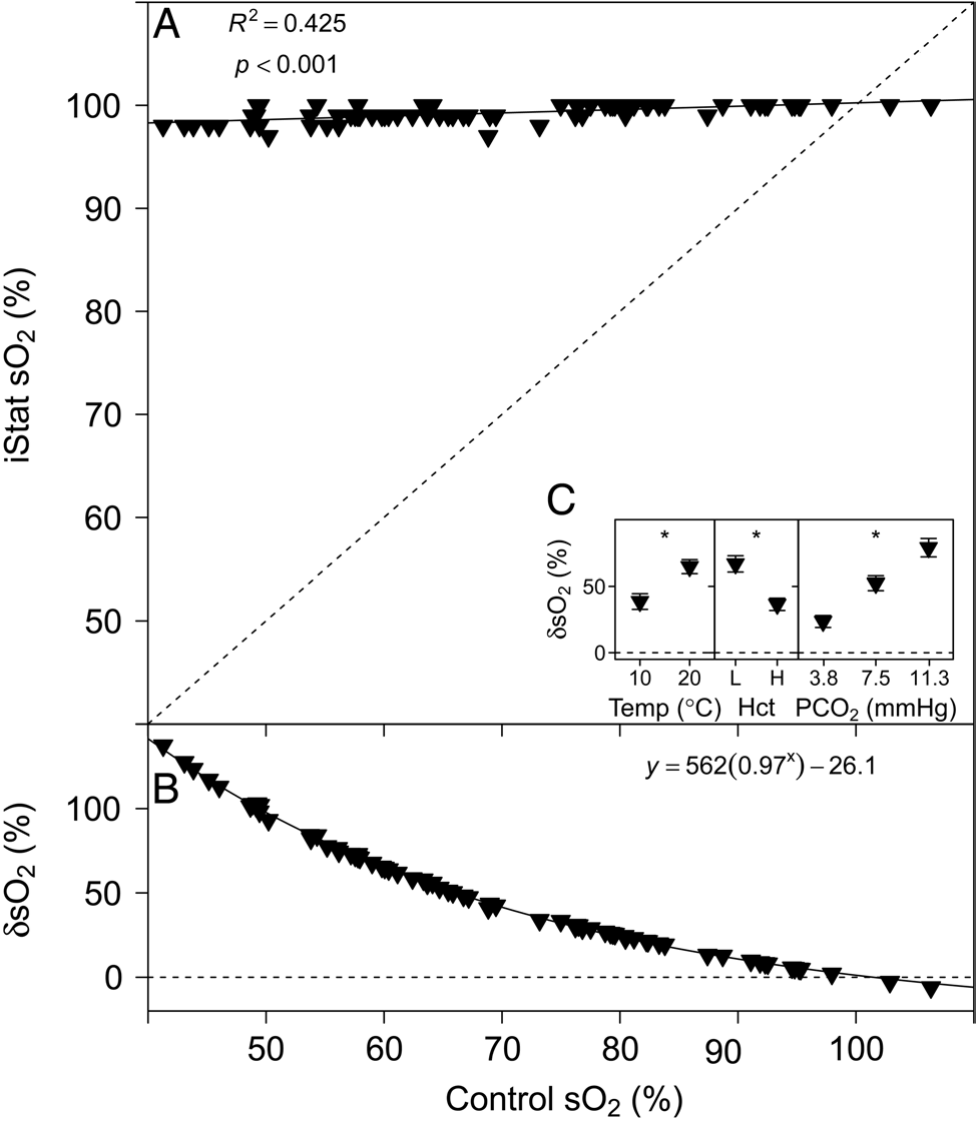
(**A**) Measurements of haemoglobin saturation (sO_2_) with the i-STAT system vs. control sO_2_ calculated by the Tucker method. (**B**) The relative error of i-STAT sO_2_ measurements, δsO_2_ (expressed as a %), [calculated as: (i-STAT sO_2_ − control HCO_3_^−^)/control sO_2_ × 100] vs. control sO_2_. Continuous lines represent the fitted models and dashed lines represent the lines of identity. (**C**) Effects of temperature (in °C), Hct (L, low; H, high) and *P*CO_2_ (in mmHg) on δsO_2_ (expressed as a %). Significant effects within treatments are indicated as ‘*’ at the *P* < 0.05 level. Data are means ± SEM, and statistical analysis was performed on the absolute δsO_2_ values.

The sO_2_ measurements performed with the i-STAT are compared with the Tucker method in Fig. 6A. A highly significant linear relationship was found between i-STAT and control pH measurements (Table 1). The relative error of sO_2_ measurements performed with the i-STAT, δsO_2_ (expressed as a percentage), is compared with control measurements in Fig. 6B and was best described by an exponential model (linear AIC, 515.73; logarithmic AIC, 419.20; and exponential AIC, 236.87), with the parameter estimates given in Table 1. Temperature (*P* < 0.001), Hct (*P* < 0.001) and *P*CO_2_ had a significant effect on δsO_2_ (*P* < 0.001; Fig. 6C).

## Discussion

The measurement of rainbow trout whole-blood pH with the i-STAT system yielded accurate and precise results over the physiological pH range. The relative measurement error (δpH) over this range was within ∼2% of control measurements performed with a thermostated pH electrode (Fig. 1B). Harrenstien *et al.* (2005) have previously described i-STAT measurement errors for pH of −5% in black rockfish (*Sebastes melanops*). Gallagher *et al.* (2010) used the i-STAT on elasmobranchs and found similar measurement errors for pH of −3.8 and −4.4% for sandbar sharks and smooth dogfish, respectively (calculated values at pH 7.5 based on the described linear relationships between control pH and i-STAT pH). Temperature had no significant effect on i-STAT pH measurements; thus, the integrated temperature correction algorithm seems to account adequately for the effects of a closed system temperature change within the cartridge (Malte *et al.*, 2014). Haematocrit did not have a significant effect on i-STAT pH measurements, which suggests that any differences in RBC size or nucleation state should not affect pH measurements. In addition, no significant effect of *P*CO_2_ was detected on i-STAT pH measurements.

In contrast, the fairly basic Hct measurement performed by the i-STAT system was consistently and significantly lower than the values measured with the standard microcapillary tubes (Fig. 2A). Over the range of tested Hct values (20–30%), measurements performed with the i-STAT system were typically 40% lower than control values (Fig. 2B); this is in line with previous studies. DiMaggio *et al.* (2010) described a similar measurement error for i-STAT Hct of −44.5% in semiole killifish. Harrenstien *et al.* (2005) found a measurement error of −30% in black rockfish, attributing this bias to differences in RBC shape between humans and fish.

The i-STAT system measures Hct indirectly by means of whole-blood conductometry (Table 2). While plasma itself is relatively conductive to electrical currents, RBCs are not; therefore, a relationship exists between the fraction of volume that is occupied by RBCs (i.e. Hct) and the conductivity of a whole-blood sample. In the i-STAT system, corrections are applied for sample temperature and sample volume (by first measuring the conductivity of a calibrant within the cartridge) and for plasma conductivity (by measuring Na^+^ and K^+^ concentrations; i-STAT Technical Bulletin, 2011, 2013a, e). All these factors are integrated into the calculation of Hct using an algorithm designed for human blood. As opposed to human RBCs, fish RBCs are nucleated, and therefore larger and different in shape (elliptical in fishes vs. double concave in humans) for a given Hb concentration (Nikinmaa, 1990). Considering the higher metabolic activity, differences in Hb isoforms and membrane composition, differences in RBC conductivity are possible. Samples measured at 10°C had a significantly lower δHct than samples measured at 20°C; thus, higher temperatures seem to have a negative effect on the accuracy of Hct measurements with the i-STAT on fish whole blood; this may be due to a temperature effect on membrane fluidity and therefore conductive properties of RBCs. In addition, Na^+^ concentration (which is integrated into Hct calculations) was not reliably measured in fish plasma.

**Table 2:** Measured and calculated blood parameters reported by the i-STAT system with CG8+ cartridges

Parameter	Method^a^
pH	Direct potentiometry
Hct	Whole-blood conductometry
Hb	Calculated from Hct
Na^+^	Ion-selective electrode potentiometry
*P*O_2_	Amperometry
*P*CO_2_	Direct potentiometry
HCO_3_^−^	Calculated from pH and *P*CO_2_
sO_2_	Calculated from *P*O_2_, pH and HCO_3_^−^

Abbreviations: Hb, haemoglobin; Hct, haematocrit; *P*CO_2_, partial pressure of CO_2_; *P*O_2_, partial pressure of O_2_; and sO_2_, haemoglobin saturation.

^a^Retrieved from the i-STAT Technical Bulletin (2013a, b, c, d, e).

The plasma of fish living in dilute freshwaters typically has ion concentrations that are lower than those found in humans (Lago *et al.*, 2008). Although the i-STAT system corrects conductivity readings for differences in plasma Na^+^ and K^+^ concentrations, no other ions are taken into account, and their concentrations may affect plasma conductivity and thus the accuracy of Hct measurements (i-STAT Technical Bulletin, 2011). Furthermore, i-STAT Hct is sensitive to the concentration of other non-conductive elements in the plasma, i.e. proteins and lipids. At Hct levels <40% and total protein concentrations <6.5 g/dl, measurements of conductivity will lead to an underestimation of Hct (i-STAT Technical Bulletin, 2011). Haematocrit levels in the present study were <30%, and total protein levels found in rainbow trout are ∼2.1 g/dl (Milligan and Wood, 1982). A significant effect of Hct on δHct indicates that having more RBCs in the blood sample will increase the accuracy of i-STAT Hct measurements; this may suggest that the confounding effects are due to differences in plasma conductivity between humans and fish, as opposed to differences in RBC conductivity. The strong Bohr/Haldane effect found in trout and the associated RBC swelling upon acidification and H^+^ buffering by Hb (Jensen, 2004) did not seem to affect i-STAT Hct measurements, because *P*CO_2_ did not have a significant effect on δHct.

The present results support previous findings that the i-STAT system underestimates Hct in fish blood; this bias was sensitive to temperature and Hct, and may be sensitive to plasma composition. Therefore, correction of i-STAT Hct measurements on fish blood using conversion factors is not recommended. If the nucleated state of fish RBCs contributed to an inaccurate Hct assessment by the i-STAT, we suspect that similar if not identical problems may be encountered with any blood that has nucleated RBCs. This would bode poorly for the majority of vertebrate animals, because over half of the species are fishes, ∼40% comprise the amphibians, reptiles and birds, while <10% are mammals that have non-nucleated erythrocytes.

Plasma Na^+^ concentration was assessed over the range of 110–150 mm. All fish were housed in the same conditions, and blood samples were pooled from several fish; therefore, initially, all samples had similar plasma Na^+^ concentrations. However, to obtain different Hct levels, samples were diluted with plasma obtained by caudal puncture. During such stress, rainbow trout RBCs will actively take up Na^+^ by β-adrenergically activated sodium-proton-exchangers (β-NHE) activity, thereby lowering plasma Na^+^ levels (Borgese *et al.*, 1987; Nikinmaa, 1990). Given that the plasma for dilution was frozen and stored for several days, no direct effect of catecholamines on blood samples was expected (Caldwell *et al.*, 2006); the relatively large range in plasma Na^+^ concentrations can be attributed to our procedures rather than the variability in the fish that were sampled. Yet, the range of plasma Na^+^ concentrations is physiologically representative for freshwater fishes housed in dilute water (Vancouver tap water, [Na^+^] 60 mm; Fu *et al.*, 2010). Over this range, no significant relationship was detected between i-STAT Na^+^ and control Na^+^ measurements with a flame photometer (Fig. 3A). The observed measurement error, δNa^+^, decreased from about +20% at 110 mm to −10% at 150 mm Na^+^, following an exponential relationship (Fig. 3B). Surprisingly, temperature had a significant effect on δNa^+^, and a non-significant trend (*P* = 0.054) suggests that Hct may affect i-STAT Na^+^ measurements outside the Hct range studied here. The i-STAT system measures Na^+^ by ion-selective potentiometry (Table 2); a calibrant solution is measured as reference, and the results are calculated based on the Nernst equation (i-STAT Technical Bulletin, 2013e). This method may be sensitive to the concentration of other ions of similar charge or size. Most fish are ammoneotelic. Thus, the relatively high concentrations of NH_4_^+^ in the plasma (0.1–0.8 mm NH_4_^+^; Wright and Wood, 1985; McDonald and Milligan, 1992) are a candidate to cause interference with i-STAT Na^+^ measurements; this possibility, however, requires further testing. Previous studies have found the i-STAT to underestimate Na^+^ concentration by −26% in semiole killifish (at 180 mm Na^+^; DiMaggio *et al.*, 2010) and by −8% in marine black rockfish (at 170 mm Na^+^; Harrenstien *et al.*, 2005). The latter findings are similar to the present results and, according to the exponential model fitted to the data, δNa^+^ at 170 mm would be −12% (Fig. 3B). Our results suggest that i-STAT measurements of Na^+^ in fish whole blood are most accurate around 140 mm, but at lower or higher concentrations the results become unreliable; the cause for this bias remains unclear. Therefore, and due to the significant effect of temperature on δNa^+^, we do not recommend using conversion factors to correct i-STAT Na^+^ measurements.

For the tested range of *P*CO_2_ values (3.8–11.3 mmHg), measurements performed with the i-STAT system were generally higher than set control values (Fig. 4A). At 3.8 mmHg, the i-STAT overestimated *P*CO_2_ by 40%. Gallagher *et al.* (2010) have described the i-STAT to overestimate *P*CO_2_ by more than 350% in sandbar sharks and smooth dogfish (at 3.5 mmHg). The results of Harrenstien *et al.* (2005) had an error closer to the present study, with the i-STAT overestimating *P*CO_2_ by 67% in black rockfish (at 10 mmHg). The significant effect of *P*CO_2_ on the measurement error, δ*P*CO_2_, suggests that the accuracy of i-STAT *P*CO_2_ measurements is highly dependent on *P*CO_2_ itself (Fig. 4C), and accuracy improves at higher *P*CO_2_ tensions. In fact, the exponential relationship between δ*P*CO_2_ and control *P*CO_2_ suggests that above 19 mmHg *P*CO_2_, δ*P*CO_2_ is <1% (Fig. 4B). Therefore, it seems that the i-STAT system can measure *P*CO_2_ accurately at higher *P*CO_2_ levels, such as expected for humans; however, measurements in the *P*CO_2_ range of most fishes (arterial *P*CO_2_ in rainbow trout, ∼2.6 mmHg; Lessard *et al.*, 1995; Brauner and Randall, 1998) will be associated with a high and potentially variable measurement error. The i-STAT measures *P*CO_2_ by direct potentiometry, and the final result is calculated based on the Nernst equation, after measuring a calibrant with known CO_2_ content as a reference (Table 2). Although the reportable range for *P*CO_2_ in the i-STAT is given as 5–130 mmHg, it seems that the low values typically present in fish cannot be measured accurately.

Bicarbonate values assessed with the i-STAT system on whole blood of rainbow trout were significantly higher than control values (Fig. 5A). The δHCO_3_^−^ for the i-STAT system was ∼20% at 8 mm HCO_3_^−^ and decreased towards higher HCO_3_^−^ concentrations (Fig. 5B). This is lower than the −47% (at 13 mm HCO_3_^−^) reported by Harrenstien *et al.* (2005). The i-STAT system calculates HCO_3_^−^ from measured pH and *P*CO_2_ values (Table 2). Therefore, it is not surprising that the *P*CO_2_ level had a significant effect on δHCO_3_^−^, which corresponds with the effect of *P*CO_2_ on δ*P*CO_2_ reported above (Fig. 4C). Both pH and *P*CO_2_ measurements in the i-STAT system are temperature corrected, and no significant effects of temperature were detected on these measurements. However, a significant effect of temperature on δHCO_3_^−^ indicates that the algorithm used by the i-STAT to calculate HCO_3_^−^ concentration is not representative for the effect of temperature on HCO_3_^−^ concentration in fish plasma (i-STAT Technical Bulletin, 2013b).

Partial pressure of O_2_ in this study was assessed with a single level, to keep the number of factor combinations manageable. Over all treatments, mean *P*O_2_ measured with the i-STAT was 77.9 ± 4.0 mmHg (at a set *P*O_2_ of 45.9 mmHg), and the mean measurement error, δ*P*O_2_, was 69.6 ± 8.6%. This measurement error for i-STAT *P*O_2_ is similar to the previously described 51.8% for sandbar sharks and 77.4% for smooth dogfish (Gallagher *et al.*, 2010). The i-STAT measures *P*O_2_ amperiometrically (Table 2), with a platinum cathode behind a gas-permeable membrane. This method is temperature sensitive, and the i-STAT technical documentation warns not to cool blood samples before measurement, because this may lead to an overestimation of *P*O_2_ (i-STAT Technical Bulletin, 2013d). The present results describe a significant effect of temperature on δ*P*O_2_, and *P*O_2_ measurements with the i-STAT were more accurate at 20°C (mean δ*P*O_2_ = 17.9 ± 4.4%) than at 10°C (mean δ*P*O_2_ = 121.3 ± 11.3%). Given that the upper lethal temperature of rainbow trout is 23–25°C (Black, 1953), accurate *P*O_2_ measurements within the physiological temperature range of this species do not seem possible with the i-STAT system. Low *P*CO_2_ levels also decreased the accuracy of i-STAT *P*O_2_ measurements, and a significant effect of *P*CO_2_ on δ*P*O_2_ was detected. Whether *P*CO_2_ affects i-STAT *P*O_2_ measurement directly or via changes in pH is unclear from the present data. At typical arterial *P*CO_2_ levels in trout (∼2.6 mmHg; Lessard *et al.*, 1995), the i-STAT system does not yield satisfactory accuracy for the measurements of *P*O_2_ (δ*P*O_2_ > 67.1 ± 12.4%).

Results for the calculated parameter sO_2_ yielded a highly significant linear relationship between i-STAT sO_2_ and control sO_2_ obtained with the Tucker method (Fig. 6A). The control values obtained in the present study are considered representative for rainbow trout whole blood, as substantiated by previous studies that have used the same tonometry method under similar conditions of *P*O_2_, *P*CO_2_ and temperature (Rummer, 2010; Rummer and Brauner, 2011). However, for the entire range of control sO_2_ values (40–100%) the i-STAT system reported sO_2_ values of 97–100%, i.e. full Hb saturation. The algorithm that the i-STAT system uses to calculate sO_2_ (Table 2) is based on a human oxygen equilibrium curve and assumes a constant Hct and ‘normal’ Hb affinity for O_2_, i.e. it does not account for inter-specific differences in Hb–O_2_ affinity, Hb isoform multiplicity, the effect of organic phosphates on Hb–O_2_ affinity or the presence of dysfunctional Hb species (carboxy-, met- and sulf-Hb; i-STAT Technical Bulletin, 2013d). While many of these factors may be relatively predictable and constant in human blood, in fishes this is not the case (Nikinmaa, 1990; Jensen *et al.*, 1998), and inter-specific variability, differences in environmental conditions and differences in the physiological state of the animal may significantly bias sO_2_ measurements performed with the i-STAT. In addition, some teleost fish species, such as trout, exhibit a strong Bohr/Root effect (Bohr *et al.*, 1904; Root, 1931), which is clearly not accounted for in the algorithm for the calculation of sO_2_ used by the i-STAT system. In trout, a higher *P*CO_2_ tension will decrease sO_2_ due to the combined effects of acidification (hence increase in H^+^ concentration) and due to the increase in CO_2_ concentration. Both H^+^ and CO_2_ can bind to Root-effect Hb and will lead to a change in conformation that stabilizes the deoxygenated state and hence leads to lower sO_2_ values at the same *P*O_2_ (Brittain, 2005). Human Hb has a moderate Bohr effect and no Root effect; therefore, changes in pH or *P*CO_2_ do not affect sO_2_ to the same degree as in fish. In the i-STAT, this is reflected in the significantly higher δsO_2_ at higher *P*CO_2_ tensions, despite a more accurate measurement of *P*O_2_ at higher *P*CO_2_ tensions. The measurement of i-STAT sO_2_ was significantly more accurate at higher Hct levels and at lower temperatures. Given that the calculation of sO_2_ is a function of *P*O_2_, it was expected that factors that would lead to an inaccurate determination of *P*O_2_ would equally be reflected in an inaccurate calculation of sO_2_. This was not the case, and the significant temperature and *P*CO_2_ effects were opposite to those effects observed for *P*O_2_. Although unexpected, this emphasizes the complex interactions that must exist between factors and provides a warning against using simple linear relationships for correction of i-STAT results. Since the temperature correction algorithm of the i-STAT system is based on human blood characteristics, using this function may increase the measurement error beyond that caused by a closed system temperature change (i.e. heating the sample to 37°C). However, Gallagher *et al.* (2010) manually temperature corrected raw values generated by the i-STAT using previously derived equations (Mandelman and Skomal, 2009) and, likewise, concluded that the i-STAT was unable to determine sO_2_ reliably in the studied elasmobranch species, suggesting that the problem goes beyond mere differences in temperature [i-STAT raw values for pH, *P*CO_2_ and *P*O_2_ were reliably corrected to 25°C using the equations derived by Mandelman and Skomal (2009)]. The characteristics of Hb in humans and fish are fundamentally different (Jensen *et al.*, 1998); therefore, the i-STAT (and algorithms based on human constants) cannot simply yield representative results for sO_2_ in fish.

### Conclusion

Measurements of pH were accurate under the tested conditions, and the i-STAT is considered an appropriate tool for assessing the acid–base status of blood in rainbow trout. Haematocrit was consistently lower than control values, but significant effects of temperature, Hct and plasma composition on the accuracy of i-STAT Hct measurements would make correction of results complex. The accuracy of i-STAT measurements of plasma Na^+^ concentration, *P*CO_2_, HCO_3_^−^ and *P*O_2_ was dependent on the measured range and associated with a high measurement error at those values typically expected for rainbow trout. In agreement with previous work, measurements of sO_2_ with the i-STAT system were not meaningful on rainbow trout whole blood, presumably due to large differences between the characteristics of Hb in humans and fish. Given the large number of studies that routinely use portable clinical analysers (including the i-STAT system) to measure blood parameters on a variety of species, the present results emphasize the need for a thorough validation of these devices within the specific study conditions. Our results indicate that, for the accurate measurement of most blood parameters in fishes (save for pH), there is no alternative to the time-consuming but well-validated laboratory methodology.
